# Virtual Imaging for a Complex Case of Previous Coarctation Repair

**DOI:** 10.1016/j.atssr.2022.10.002

**Published:** 2022-10-13

**Authors:** Yuji Matsubayashi, Kenichi Kamiya, Tomoaki Suzuki, Shunta Miwa, Yotaro Mori, Masahide Enomoto, Naoshi Minamidate, Noriyuki Takashima

**Affiliations:** 1 Department of Cardiovascular Surgery, Shiga University of Medical Science, Setatsukinowa-cho, Otsu, Shiga, Japan

## Abstract

Repeated surgical procedures for previous aortic coarctation is challenging. Three-dimensional imaging may be useful to visualize the complex anatomy more clearly than with computed tomography imaging, possibly leading to better outcomes. To evaluate the feasibility and efficacy of virtual imaging for preoperative planning of thoracic aortic surgery in this case, we used Vesalius 3D software, 3-dimensional image processing software, and detailed anatomic exploration. This technology may serve to optimize anatomically complex thoracic aortic surgery.

True aneurysm formation after patch repair of coarctation of the aorta has been known as a major long-term complication.^1^ Surgical intervention is mostly difficult owing to severe adhesions and fragility of these aneurysms.^1^ We present Vesalius 3D software, 3-dimensional (3D) image processing software, to optimize the surgical procedure for a complicated and challenging case. The 3D virtual imaging system enables us to explore the patient’s unique anatomy quickly, intuitively, and in clear detail, allowing prompt, easy, and accurate preoperative planning.^2^

A 47-year-old woman underwent patch repair for simple postductal aortic coarctation (Figures 1A, 1B). Thirty years later at the age of 77 years, the patient was referred to our hospital with a diagnosis of expanding aortic aneurysm in the thoracic distal arch. Preoperative computed tomography angiography (CTA) revealed a saccular aneurysm with a maximum diameter of 57 mm, just distal to the left subclavian artery, and surgical reintervention was indicated (Figure 1C). Endograft therapy was considered unsuitable because of the narrow proximal landing zone (15 mm), whereas the distal diameter was 27 mm at the level of T6. Repeated thoracotomy was required, and the risk of repeated surgical procedures was high. The expected operative risk factors were the innate vascular fragility due to aortic coarctation, anatomic complexity, and postoperative adhesions. Elaborate preoperative planning was indispensable before open repair.

**Figure 1:**
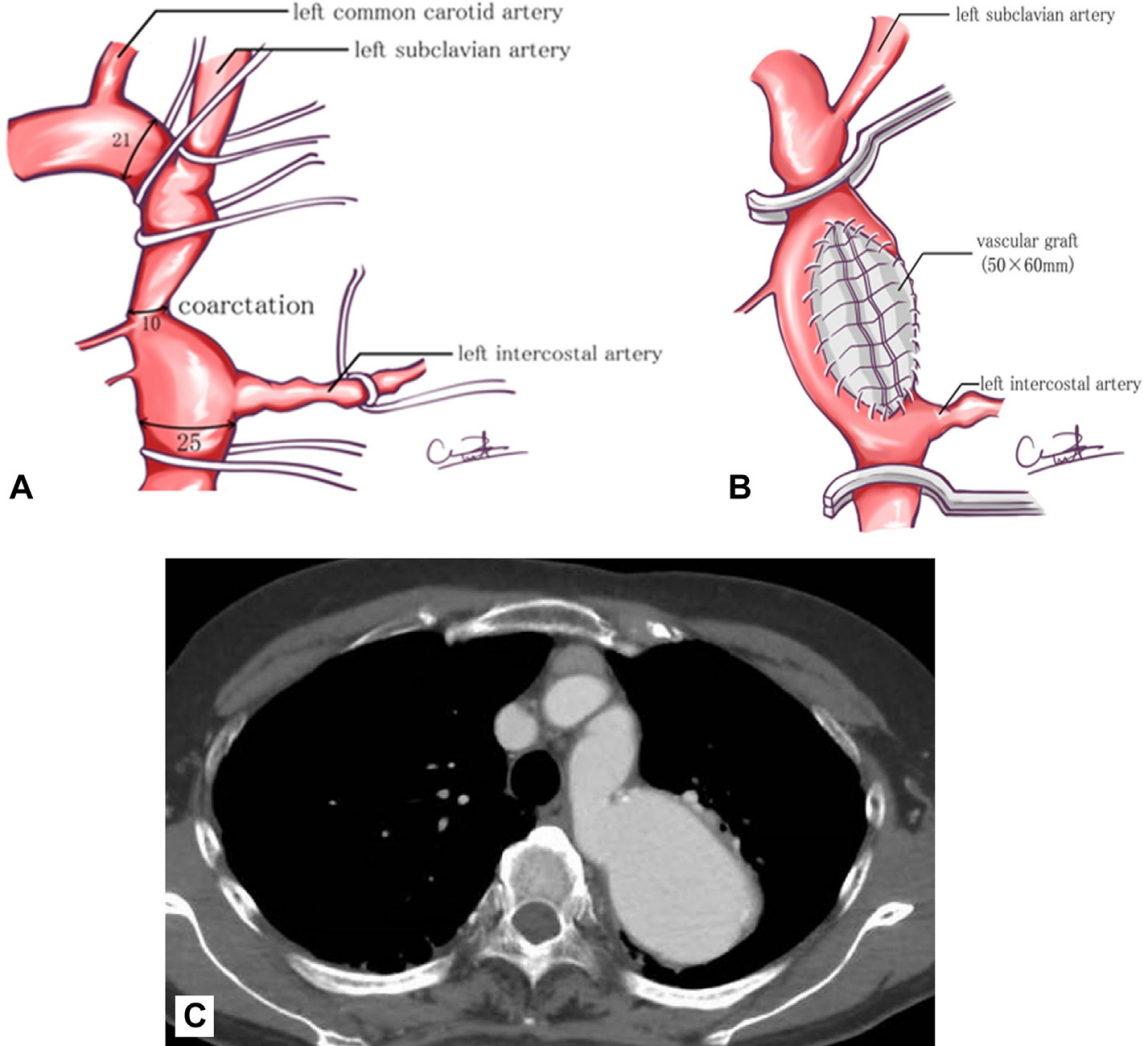
(A) Previous operation record. (B) Aortic patch plasty with a trimmed vascular graft. (C) Preoperative computed tomography image. The saccular aneurysm with a maximum diameter of 57 mm just distal to the left subclavian artery is shown.

We planned the most suitable operative strategy based on conventional CTA; however, we also processed the CTA images using virtual reality software (Vesalius 3D; PS Medtech) to reconstruct 3D images of the thoracic aortic anatomy (Supplemental Video). The procedure was performed through left posterolateral thoracotomy at the fourth intercostal space, and cardiopulmonary bypass was established using left femoral artery and vein cannulation (Figure 2A). An open proximal anastomosis was performed with a Dacron tube graft (24-mm J-Graft Shield Neo; Japan Lifeline Co Ltd) using 3-0 polypropylene sutures with a polyester felt strip under hypothermic arrest at a core temperature of 24 °C (Figures 2B-2D). During circulatory arrest, continuous blood flow was maintained from the femoral arterial cannula to prevent air embolization (Figure 3A). The distal anastomosis was completed during rewarming (Figure 3B). The proximal anastomosis under hypothermic arrest required 7 minutes. The aortic cross-clamping time was 51 minutes, and the cardiopulmonary bypass time was 129 minutes. The postoperative course was uneventful with no complications (Figures 3C, 3D).

**Figure 2:**
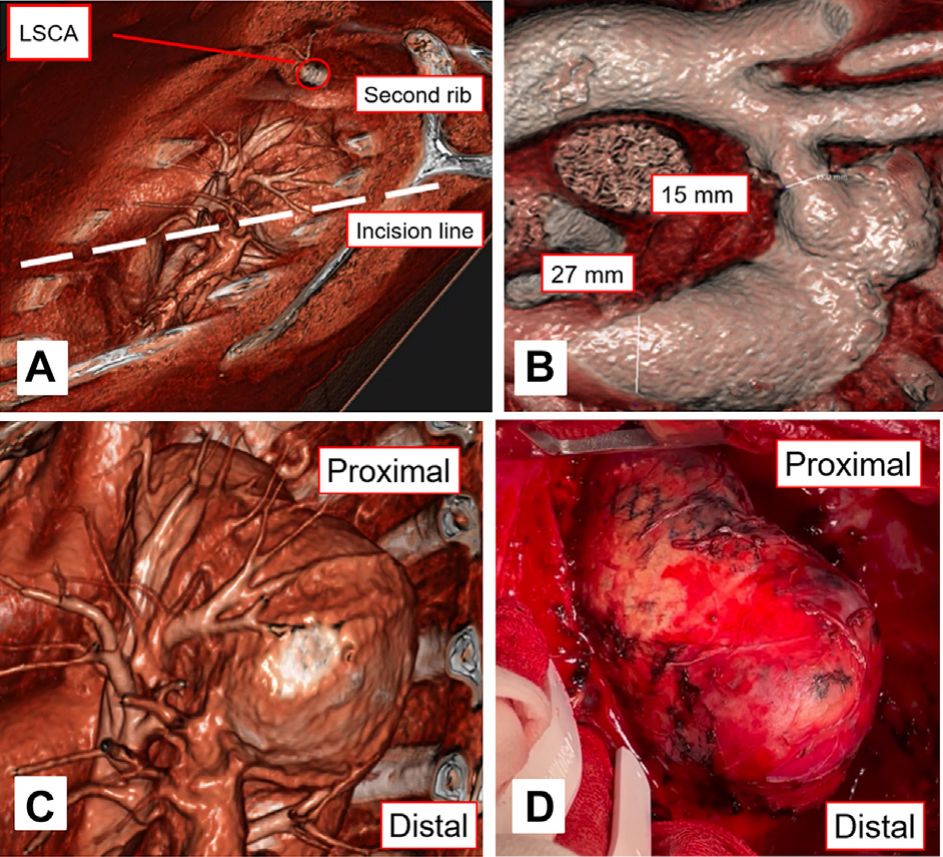
(A) Actual surgical approach. The incision line and the patient’s posture were decided on the basis of the surgeon’s approach angle, as observed with virtual reality. (B) Discrepancy between proximal diameter and distal diameter. (C) Virtual reality imaging. (D) The aneurysm matched the virtual reality image. The aneurysm had arisen from the other side of the previous patch. (LSCA, left subclavian artery.)

**Figure 3:**
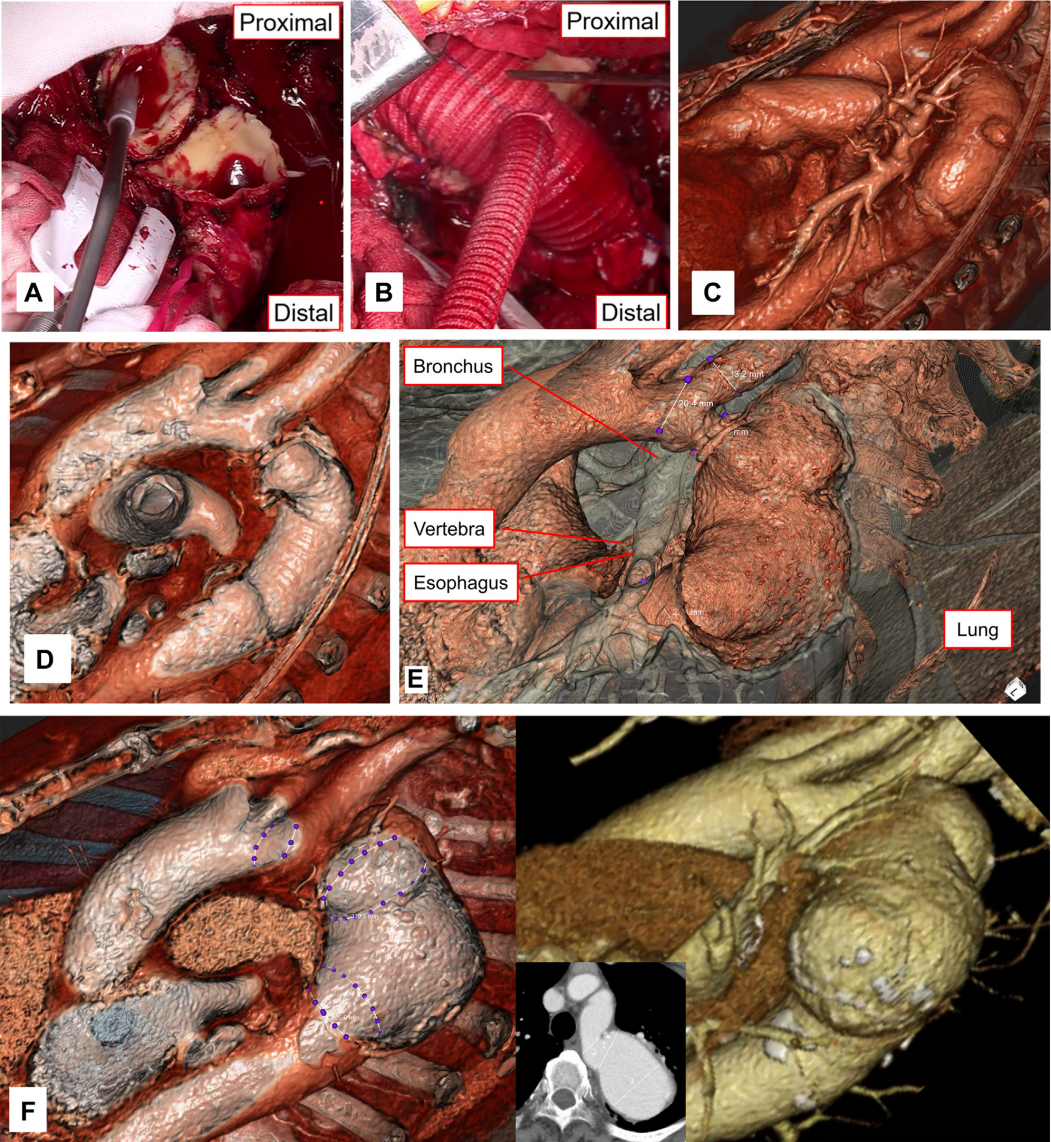
(A) Open proximal anastomosis. (B) The aneurysm was replaced with a prosthetic graft. (C) Postoperative virtual reality image. (D) Postoperative virtual reality image showing the inside of the anastomosis and the prosthetic graft. (E) Extraluminal components, such as the bronchi, vertebra, lungs, and esophagus, can be visualized by adjusting the window level. (F) Side-by-side virtual 3-dimensional image and conventional volume-rendered image. The circumference of the aorta can be measured intuitively, directly, and easily by placing dots on the vessel wall on virtual 3-dimensional images. Inset: The distance cannot be measured unless a certain cross section is decided on conventional volume-rendered image.

## Comment

A few decades ago, Dacron patch aortoplasty was a standard therapy for aortic coarctation.^3^ However, a relatively high incidence of aneurysm formation after aortic patch repair has been reported, ranging from 5% to 38%, with aneurysmal dilation progressing within 6 to 18 years.^1^ The high incidence of rupture during reoperation is reportedly due to the thin and fragile aneurysm wall. Thus, a safe method must be considered to avoid hemorrhagic disaster.^1^

CTA has been proven to be excellent for the diagnosis of thoracic aortic aneurysm. Virtual 3D images show the target region in exquisite detail, revealing the intravascular structures of both the proximal and distal sites for anastomosis, the morphologic features of the cervical vessels, and the surrounding structures of the descending aorta, such as the intercostal arteries, left bronchi, and esophagus.^4^ In this case, extraluminal components, such as the bronchi, vertebra, lungs, and esophagus, can be visualized by adjusting the window level (Figure 3E). It is also possible to realize the anatomic relationship between the aorta and extraluminal tissues from any angle. The intraluminal structure can also be visualized, which can help to grasp the depth and expanse of the anterior aortic wall to the posterior aortic wall. Thus, the operator can imagine the precise internal structure of the aorta without opening the aortic wall before the surgical procedure (Supplemental Video).

To realize the 3D anatomic structure, 3 cross sections in the horizontal, coronal, and sagittal views are required with conventional CTA, and surgeons need to reconstruct these images in their mind, which is not always precise. As a solution to this problem, our technology provides an accurate 3D anatomy, which makes it possible for all surgeons to acquire the same 3D view.^5^

With conventional CTA, the diameter of the aorta is measured only on a limited cross section. Volume-rendered images reconstructed from CTA are not suitable for measuring distance; however, the circumference of the aorta can be measured intuitively and easily by placing dots on the vessel wall on virtual 3D images (Figure 3F).

In conclusion, the 3D virtual imaging system used in this study enabled us to explore the patient’s unique anatomy quickly, intuitively, and in clear detail, allowing prompt, easy, and accurate preoperative planning.^2^ This technology could provide outstanding assistance to optimize anatomically complicated thoracic aortic surgery.

## References

[1] Roth M., Lemke P., Schönburg M., Klövekorn W.P., Bauer E.P. (2002). Aneurysm formation after patch aortoplasty repair (Vossschulte): reoperation in adults with and without hypothermic circulatory arrest. Ann Thorac Surg.

[2] Kamiya K., Nagatani Y., Matsubayashi Y. (2021). A virtual-reality imaging analysis of the dynamic aortic root anatomy. Ann Thorac Surg.

[3] Beckmann E., Jassar A.S. (2018). Coarctation repair—redo challenges in the adults: what to do?. J Vis Surg.

[4] Kamiya K., Matsubayashi Y., Terada S. (2022). Ex-vivo aortic root and coronary artery cast measurement to validate the accuracy of virtual imaging. J Card Surg.

[5] Kamiya K., Nagatani Y., Terada S. (2022). Validation of virtual imaging of a dynamic, functioning aortic valve using an ex-vivo porcine heart. Ann Thorac Surg.

